# Thermal Safety of Endoscopic Usage in Robot-Assisted Middle Ear Surgery: An Experimental Study

**DOI:** 10.3389/fsurg.2021.659688

**Published:** 2021-05-14

**Authors:** Jinxi Pan, Haoyue Tan, Jun Shi, Zhaoyan Wang, Olivier Sterkers, Huan Jia, Hao Wu

**Affiliations:** ^1^Department of Otolaryngology-Head and Neck Surgery, Shanghai Ninth People's Hospital, Shanghai Jiao Tong University School of Medicine, Shanghai, China; ^2^Ear Institute, Shanghai Jiao Tong University School of Medicine, Shanghai, China; ^3^Shanghai Key Laboratory of Translational Medicine on Ear and Nose Diseases, Shanghai, China; ^4^APHP, Groupe Hospitalo-Universitaire Pitié Salpêtrière, Otorhinolaryngology Department, Unit of Otology, Auditory Implants and Skull Base Surgery, Paris, France

**Keywords:** endoscopic ear surgery, robot-assisted, thermal damage, endoscope, robotic

## Abstract

**Objectives:** The widespread application of endoscopic ear surgery (EES), performed through the external auditory canal, has revealed the limitations of the one-handed technique. The RobOtol® (Collin ORL, Bagneux, France) otological robotic system has been introduced to enable two-handed procedures; however, the thermal properties of dedicated endoscopes, which are usually used in neurosurgery, called “neuro-endoscopes,” have not yet been clarified for the robotic systems. In this study, we aimed to profile the thermal characteristics of two dedicated neuro-endoscopes, as compared to endoscopes used routinely in manual EES, called “oto-endoscopes,” and defined by a smaller diameter and shorter length, and to discuss the safe application of robotic assistance in EES.

**Methods:** Two neuro-endoscopes (3.3 mm, 25 cm, 0°/30°) were studied using two routine light sources (LED/xenon), and two routine oto-endoscopes (3 mm, 14 cm, 0°/30°) were initially measured to provide a comprehensive comparison. Light intensities and temperatures were measured at different power settings. The thermal distributions were measured in an open environment and a human temporal bone model of EES. The cooling measures were also studied.

**Results:** Light intensity was correlated with stabilized tip temperatures (*P* < 0.01, *R*^2^ = 0.8719). Under 100% xenon power, the stabilized temperatures at the tips of 0°, 30° neuro-endoscopes, and 0°, 30° oto-endoscopes were 96.1, 60.1, 67.8, and 56.4°C, respectively. With 100% LED power, the temperatures decreased by about 10°C, respectively. For the 0° neuro-endoscope, the illuminated area far away 1cm from the tip was below 37°C when using more than 50% both power, while this distance for 30° neuro-endoscope was 0.5 cm. In the EES temporal bone model, the round window area could reach 59.3°C with the 0° neuro-endoscope under 100% xenon power. Suction resulted in a ~1–2°C temperature drop, while a 10 mL saline rinse gave a baseline temperature which lasted for 2.5 min.

**Conclusion:** Neuro-endoscope causes higher thermal releasing in the surgical cavity of ESS, which should be especially cautious in the robotic system usage. Applying submaximal light intensity, a LED source and intermittent rinsing should be considered for the safer robot-assisted EES using a neuro-endoscope that allows a two-handed surgical procedure.

## Introduction

Endoscopic ear surgery (EES) has become a popular technique in recent years as a result of its better exposure and closer view of micro-anatomic structures in the middle ear (1, 2). However, the broad clinical applicability of EES has revealed some limitations, and one of the most frequently mentioned is the one-handed technique, which makes complex operations more challenging especially when hemorrhage occurs (1, 3, 4). Therefore, endoscope holders for endoscopic surgery (5–8) and robotic arms such as RobOtol® (Collin ORL, Bagneux, France) (9) have been introduced to enable two-handed procedures. To adapt these devices, longer endoscopes (10, 11) had to be applied in order to avoid the interference between surgeon's hands and robotic arm/ endoscopic holder. For RobOtol®, the specific endoscopes were routinely used in neurosurgical procedures (neuro-endoscopes). The usage of endoscopic holder or robotic system also changes the surgical mode for endoscope, that the endoscope stays longer and more statically in EAC. These factors might potentially bring out thermal damage to local tissue.

Several studies have reported thermal injuries caused by endoscopic application in the nasal (12–14) and abdominal cavities (15, 16). Others have investigated the thermal risk associated with the use of oto-endoscopes (17–23), such as deterioration of inner ear function (18) and facial palsy (23). Up to now, the thermal effects of neuro-endoscopes have not been investigated or published, and the RobOtol® system works with these endoscopes. Taking into consideration the thermal effects of the light source, endoscope size, power settings, and cooling mechanisms (17–22), the safety of neuro-endoscopes in EES should be thoroughly and precisely investigated, particularly those devices adapted for robotic assistance, which is considered to represent the future tendency for EES.

This study aimed to investigate the thermal effects of neuro-endoscopes, in an open environment and with EES in a human temporal bone model, as compared to usual endoscopes for manual EES, named oto-endoscopes. The effects of cooling by clinical suction and rinsing were also investigated. The aim was to provide safety information and optimum configuration for robot-assisted EES techniques.

## Materials and Methods

### Endoscope Systems

Four regular Karl Storz endoscopes were investigated: the Hopkins 28007AA (3.3 mm diameter, 0° tip, 25 cm length) and Hopkins 28007BA (3.3 mm, 30°, 25 cm), which are conventionally used in neurosurgery (named neuro-endoscopes either Neuro-0 and Neuro-30 in this study), and are now used with a robotic system dedicated for ear surgery (RobOtol®, Collin ORL, Bagneux, France), and the Hopkins 7220AA (3 mm, 0°, 14 cm) and Hopkins 7220BA (3 mm, 30°, 14 cm), which are conventionally used in otology (named oto-endoscopes either Oto-0 and Oto-30 in this study). Two endoscope systems were used: a 300 W xenon light source (Model 20133120, Karl Storz Endoskope, Tuttlingen, Germany) and a 175 W LED light source (Model 20161420, Karl Storz Endoskope), connected to the endoscope being tested using the same standard fiber optic cable (Model 495NA, Karl Storz Endoskope).

### Light Intensity and Temperature Measurements

The light intensity was measured with a lux meter (TES 1332A, TES Electrical Electronic Corp, Taiwan, PRC) with an accuracy of ±4% rdg ± 10 dgts (>10,000 lux). The temperature was measured with a JK808 eight-channel temperature tester and accessory JK80x data acquisition software (Changzhou Jin'ailian Electronic Technology Co., Ltd., Changzhou, PRC), with a measurement accuracy of 0.2% ± 1°C.

### Light Intensity and Thermal Distribution in an Open Environment

The tip of the endoscope was firmly placed in close contact with the light detector of the lux meter. Light intensity was recorded when the source output was stable. The initial power was set at 5% and then tested to 100% in 5% increments. Both light sources were studied with each of the four endoscopes.

**Figure 1:**
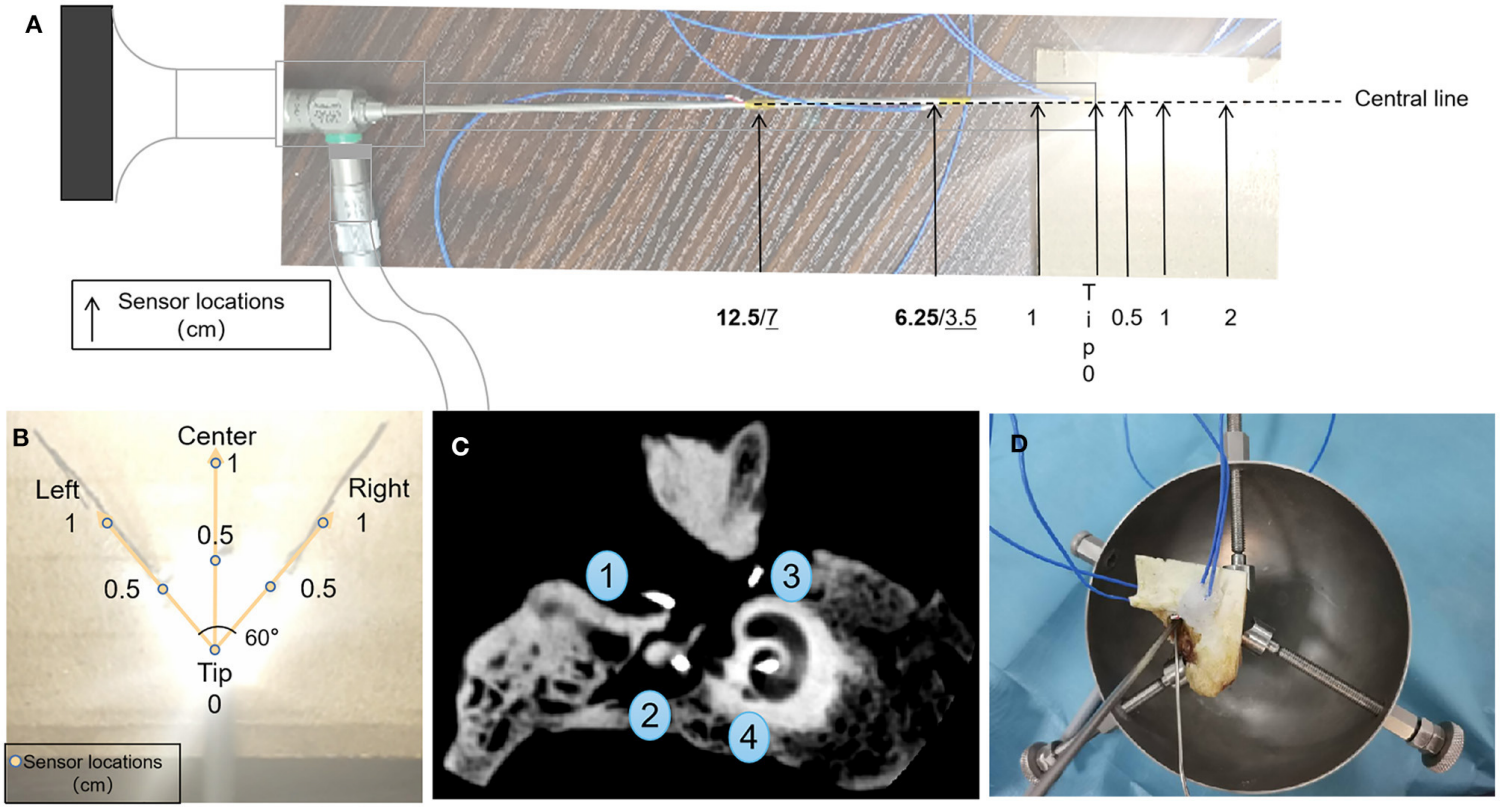
Configurations for sensors in the open environment **(A,B)** and temporal bone **(C)**, and transcanal endoscopic ear surgery settings **(D)**. **(A)** Temperature sensors placement relating to the tip of the endoscope. Boldface indicates the 1/2 shaft length and 1/4 shaft length distances (cm) of the sensors to the rear of the tip of the neuroscope, and underline indicates their placement to the rear of the tip of the oto-endoscope. **(B)** Temperature sensors placement according to the illuminated area. **(C)** Representative cone-beam computed tomography images of sensor positions in temporal bone 4R. Fundus of the external auditory canal, aditus ad antrum, round window/niche, and modiolus/fundus of the inner auditory canal.

Seven thermocouple sensors were used to measure the axial thermal distributions for each of the endoscopes. Three sensors were placed 0.5, 1, and 2 cm in front of the tip of the endoscope along the light axis, and the remaining four were placed at the tip of the endoscope, 1 cm to the rear of the tip, 1/4 shaft length rear of the tip, and 1/2 shaft length rear of the tip (Figure 1A). The sensors' data collection cycle was adjusted to 1 s. After the baseline (ambient) temperature had been recorded for 30 s, the light source for the endoscope was turned on for 600 s, then the subsequent 180 s was recorded as a cooling period after the light source had been turned off. According to the light intensity results and clinical requirements, the power was set at 20, 45, 46, 47, 48, 49, 50, 51, 52, 53, 54, 55, and 100% for the xenon light source, and at 5, 10, 15, 20, 25, 50, and 100% for the LED light source.

Seven thermocouple sensors were used to record the thermal distribution in the illuminated area. They were placed at the tip, and 0.5 cm (left, center, right), and 1 cm (left, center, right) in front of the tip. Left and right sensors were placed at an angle of 60° centered on the tip as this is the edge of the illuminated area (Figure 1B). The power settings were 50% or 100% for both light sources.

### Thermal Distribution With EES Using a Human Temporal Bone Model

Two left sides (1L; 2L) and two right sides (3R; 4R) of temporal bones (Henan Haizhirun Biotechnology Co., Ltd., Henan, PRC) were studied. Four sensors were firmly fixed at four anatomic landmarks: the fundus of the external auditory canal, the aditus ad antrum (after a canal wall-up mastoidectomy had been drilled), the round window/niche, and the modiolus/fundus of the inner auditory canal. The mastoidectomy cavity and the orifice of the internal auditory canal were sealed by bone wax (Knochenwachs; B. Braun Surgical, S.A., Barcelona, Spain) to prevent heat dissipation. Cone-beam computed tomography scans were performed to verify the correct location of these sensors (Figure 1C).

The Neuro-0 and Oto-0 endoscopes were used in this experiment, and were firmly placed at the level of the tympanic annulus (Figure 1D) via the external auditory canal (EAC), without contacting the skin. After the baseline (ambient) temperature had been recorded for 30 s, the LED or xenon light source was turned on at 100 or 50% power settings for 10 min, and subsequently, the local temperature was recorded for 3 min after turning off the light source.

The effect of cooling measures was studied by applying suction or rinsing, which are the general procedures used in routine EES, and using a xenon light source which produces more heat than the LED source. A #3 French (~1 mm) suction tube (Chong Ning Medical Co., Ltd, Shanghai, PRC) with a negative pressure of 0.04–0.05 MPa, which is regularly used in middle ear surgery, was placed close to the endoscope tip. For the continuous suction cooling test, after an initial 60 s period, suction was turned on. For the discontinuous suction cooling test, after an initial 60 s period, two cooling periods of 30 and 60 s were performed separated by a 30 s interval. The effect of rinsing measures was studied by injecting ambient temperature saline (10 mL) into the middle ear cavity through the EAC and which was immediately sucked out.

### Statistical Analysis

Light intensity (lux), power setting (%), time (s), and temperature (°C) were measured. The ambient temperature during the experiments was 25 ± 3°C. Values are presented as means and standard deviation or ratios. Statistical analyses were conducted with WPS Office (Kingsoft Office Corp., Beijing, PRC) and GraphPad Prism8 (GraphPad Prism Software, San Diego, CA, USA), with a significance level represented as ^*^P < 0.01.

This work did not contain animal/human studies. No IRB (institutional review board) approval was required.

## Results

### Light Intensity

**Figure 2:**
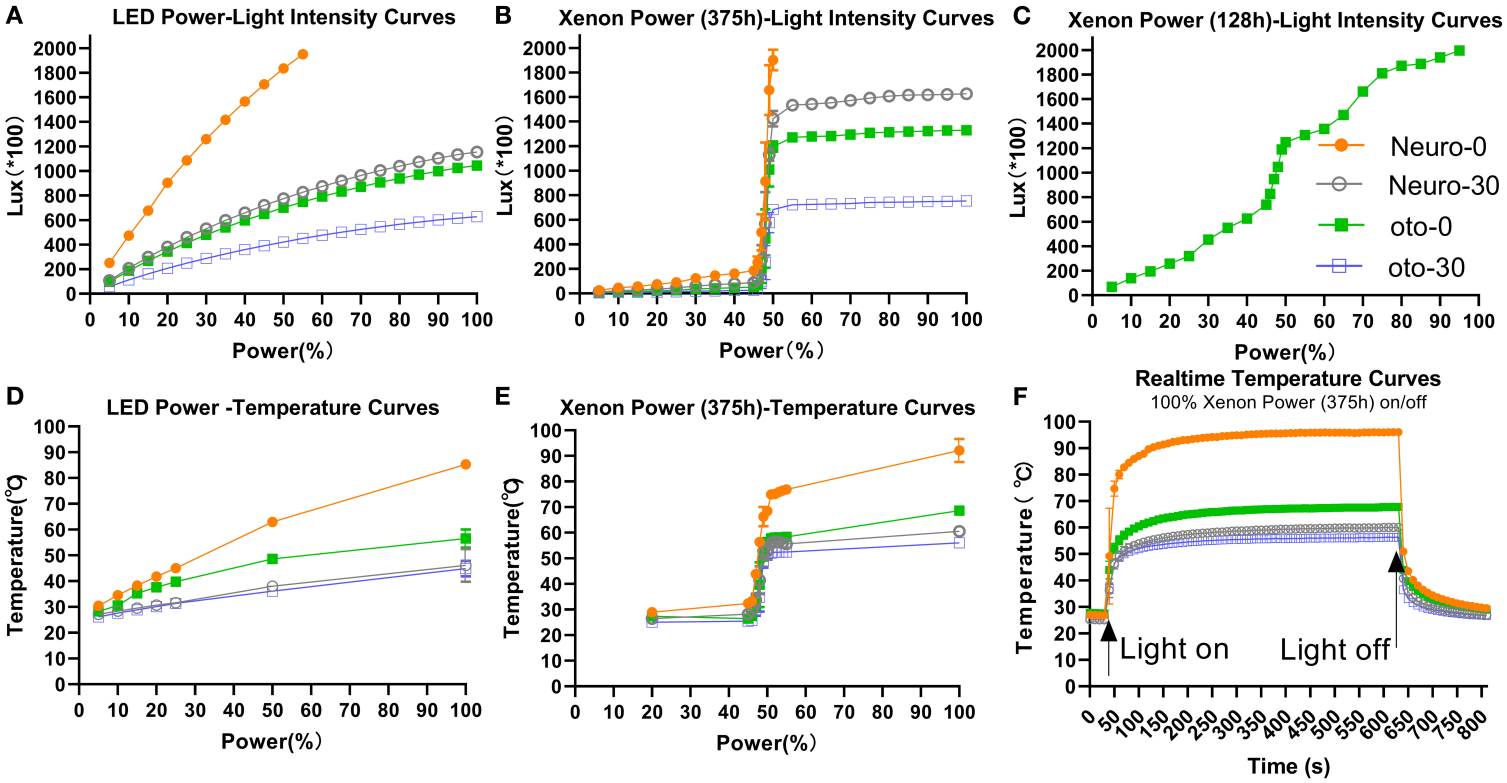
Light intensity **(A–C)** and temperature **(D–F)** changes with various endoscopes using different LED or xenon light source power settings. **(A)** The fitting equations of the power-light intensity curves are as follows: Neuro-0: y = −0.2533x^2^ + 49.16x + 9.507, *R*^2^ = 0.9999; Neuro-30: y = −0.07455x^2^ + 18.61x + 31.53, *R*^2^ = 0.9996; Oto-0: y = −0.06736x^2^ + 16.83x + 28.61, *R*^2^ = 0.9996; Oto-30: y = −0.04046x^2^ + 10.11x + 17.65, *R*^2^ = 0.9996. **(D)** The fitting equations of the power-temperature curves are as follows: Neuro-0: y = −0.002671x^2^ + 0.8610x + 25.94, *R*^2^ = 0.9980; Neuro-30: y = −0.0007388x^2^ + 0.2805x + 25.52, *R*^2^ = 0.8607; Oto-0: y = −0.003346x^2^ + 0.6454x + 25.42, *R*^2^ = 0.9732; Oto-30: y = −0.0005655x^2^ + 0.2537x + 25.13, *R*^2^ = 0.9626.

Light intensity increased with power setting (Figures 2A–C) and differed among the four endoscopes. The larger diameter and 0° endoscope had the highest light intensity. The light intensities at 5% xenon power were 25 ± 1 (^*^100 lux), 11 ± 0 (^*^100 lux), 7 ± 1 (^*^100 lux), and 4 ± 0 (^*^100 lux) for Neuro-0, Neuro-30, Oto-0, and Oto-30, respectively. The light intensities at 100% xenon power were >2,000 (^*^ 100 lux), 1,627 ± 3 (^*^100 lux), 1,330 ± 12 (^*^100 lux), and 753±6 (^*^100 lux) for corresponding endoscopes. With the LED light source (Figure 2A), the curves could be described using quadratic equations; however, with the xenon light source (with 375 h registered), the light intensity increased sharply between 45 and 50% power (Figure 2B). This phenomenon could also be observed with a newer xenon light source (with 128 h registered) (Figure 2C), but less sharply.

### Temperature at Endoscope Tips

Temperatures at the endoscope tips increased with power (Figures 2D,E) and changed rapidly over time when switching on/off power (Figure 2F). Under 100% xenon power, the stabilized temperatures of Neuro-0, Neuro-30, Oto-0, and Oto-30 endoscopes were 96.1, 60.1, 67.8, and 56.4°C, respectively (Figure 2E). Under 50% xenon power, the stabilized tip temperatures of the corresponding endoscopes were 68.5, 52.8, 56.4, and 51.1°C, respectively. But there was a sharp increase in temperature from 45 to 50% xenon power, similar to the power–light intensity curves.

The LED light source showed a more stable output than the xenon light source with increasing power. Adopting 100% LED power, the stabilized temperatures of Neuro-0, Neuro-30, Oto-0, and Oto-30 endoscopes were 86.9, 51.9, 53.3, and 47.6°C, respectively (Figure 2D). Adopting 50% LED power, the stabilized temperatures of the corresponding endoscopes were 62.9, 38.1, 48.6, and 36.1°C, respectively.

Overall, the stabilized endoscope tip temperatures and light intensities were correlated among all combinations (temperature = 0.02282^*^light intensity + 27.01, *p* < 0.01, *R*^2^ = 0.8719).

### Thermal Distribution on the Endoscope Shaft and in an Open Environment

The temperature was highest at the tip of the endoscope and gradually decreased with distance in front of or behind the tip (Tables 1, 2). At 100% xenon or LED power, the temperatures of all endoscope shafts were below 37°C, as was the temperature 0.5 cm in front of the tip of the Oto-0 and Oto-30 endoscopes. But for Neuro-0, at 50% xenon or LED power, the maximum temperature at 0.5 cm in front of the tip might still be higher than 37°C (45.4 and 38.5°C, respectively).

**Table 1:** Thermal spread with distance in front of or behind the tip and with the xenon light source.

**Distance to tip (cm)**^**a**^		**Neuro-0**		**Neuro-30**
		**100% power**	**50% power**	**100% power**
0		92.1 ± 4.5	62.9 ± 6.7	60.5 ± 1.4
−1		31.9 ± 0.8	31.1 ± 0.1	30.2 ± 0.2
+0.5	Center	46.9 ± 1.5	45.4 ± 2.0	37.4 ± 0.4
	Left	38.9 ± 1.4	38.5 ± 1.3	34.9 ± 0.5
	Right	42.5 ± 0.2	39.5 ± 1.7	35.7 ± 0.2
+1	Center	31.5 ± 0.9	32.4 ± 0.5	30.3 ± 0.2
	Left	29.0 ± 0.3	29.7 ± 0.3	28.2 ± 0.1
	Right	30.6 ± 0.2	31.5 ± 0.4	30.1 ± 0.3

a *Negative values in cm indicate measurements made on the endoscope shaft; Positive values in cm indicate those made in front of the tip in an open environment (see Figure 1)*.

*Temperature (°C, means ± standard deviation)*.

**Table 2:** Thermal spread with distance in front of or behind the tip and with the LED light source.

**Distance to tip (cm)**^**a**^		**Neuro-0**		**Neuro-30**
		**100% power**	**50% power**	**100% power**
0		85.3 ± 1.8	52.3 ± 0.3	46.2 ± 6.4
−1		34.2 ± 0.3	32.3 ± 0.1	30.7 ± 0.2
+0.5	Center	48.6 ± 1.2	38.5 ± 0.2	32.8 ± 1.2
	Left	42.0 ± 0.9	37.8 ± 1.1	32.2 ± 0.2
	Right	46.5 ± 0.1	35.5 ± 0.8	32.7 ± 0.5
+1	Center	33.4 ± 3.0	30.7 ± 0.2	28.2 ± 0.3
	Left	32.3 ± 0.3	29.9 ± 0.4	26.7 ± 0.2
	Right	34.6 ± 0.3	30.7 ± 0.5	28.2 ± 0.4

a *Negative values in cm indicate measurements made on the endoscope shaft; Positive values in cm indicate those made in front of the tip in an open environment (see Figure 1)*.

*Temperature (°C, means ± standard deviation)*.

### Characteristics of Temperature Elevation in Temporal Bone

**Figure 3:**
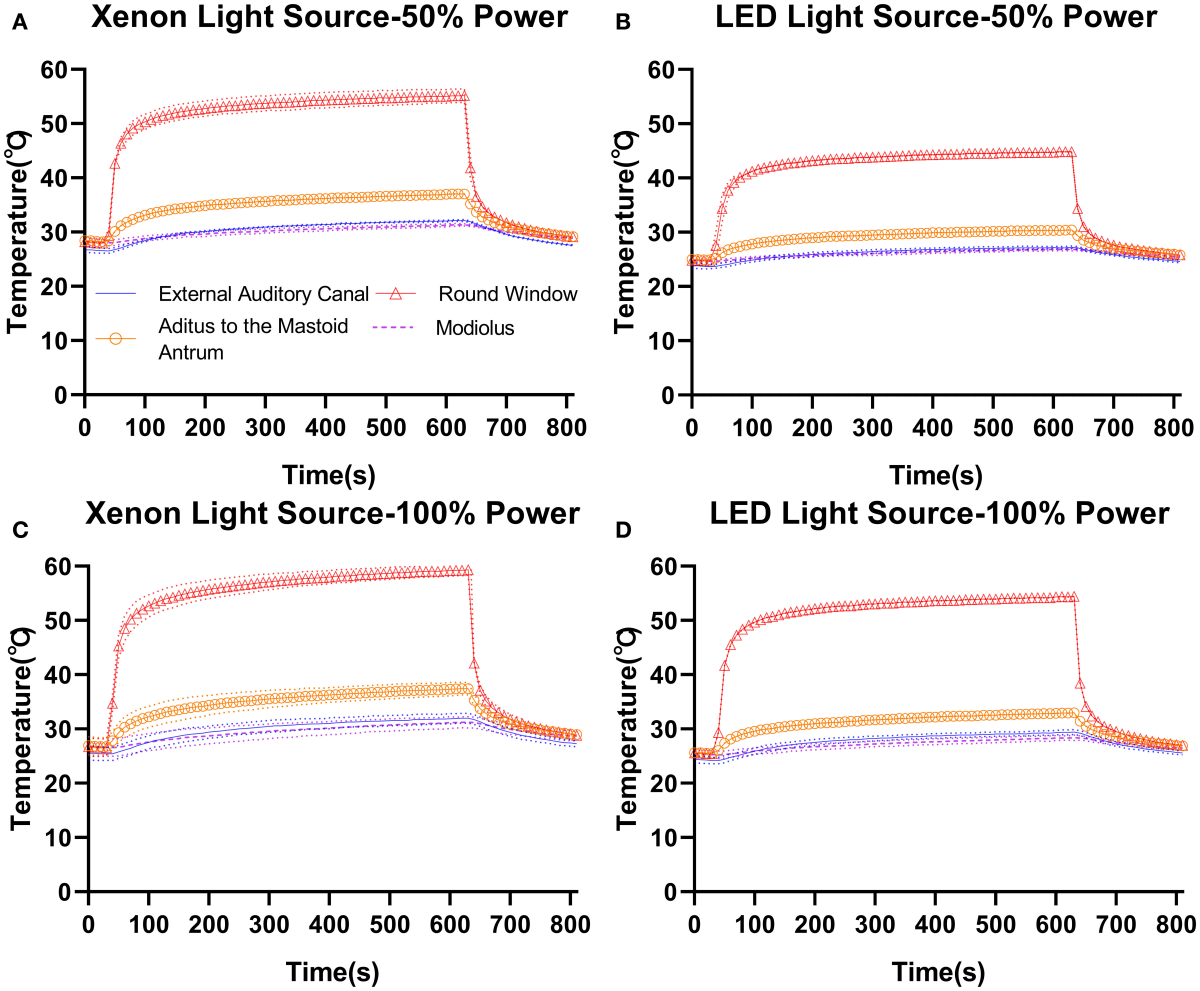
Temperature elevation in temporal bone (4R) with the Neuro-0 endoscope under different xenon **(A,C)** and LED **(B,D)** light source power settings.

Temperature elevation in temporal bone was similar between xenon (Figures 3A,C) and LED (Figures 3B,D) light source power settings. In the temporal bone, with the Neuro-0 endoscope under 100% light source power (Figures 3C,D), the temperature increased slightly at the EAC fundus (xenon: 26.1–33.7°C; LED: 29.2–32.2°C), aditus ad antrum (xenon: 28.4–37.4°C; LED: 32.7–36°C), and modiolus/fundus of the inner auditory canal (xenon: 28–38.1°C; LED: 28.2–36.4°C), except at the round window (xenon: 54.1–59.3°C; LED: 52.4–57°C), a critical location between the middle and inner ears. For the Neuro-0 endoscope, the temperature at the round window was still elevated above 37°C using the two light sources at 50% power (Figures 3A,B).

**Figure 4:**
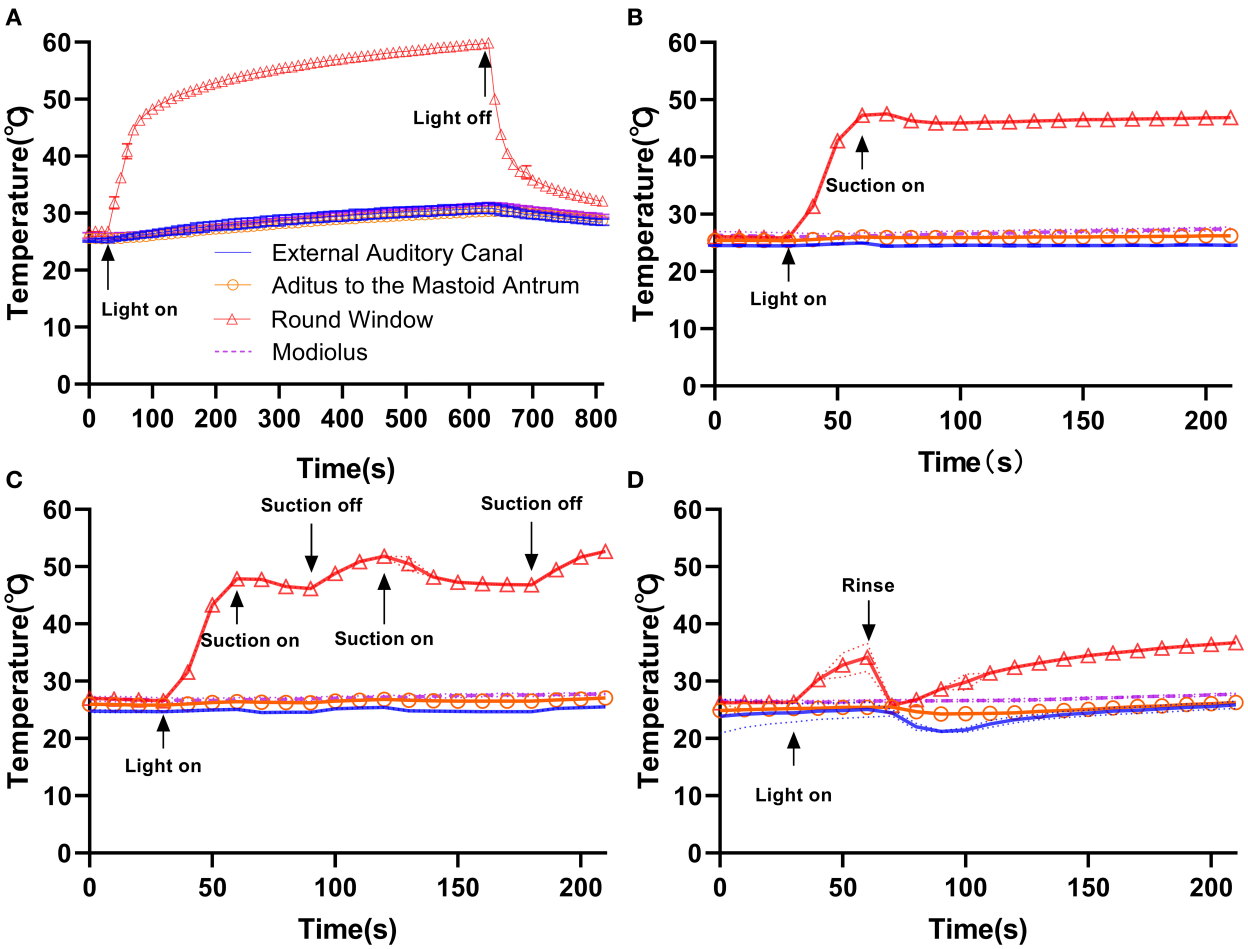
Compared with the control group **(A)**, continuous suction **(B)**, discontinuous suction **(C)**, and rinsing **(D)** resulted in different cooling effects in temporal bone with the Neuro-0 endoscope under 100% xenon light source power. Rinsing resulted in a better cooling effect.

When applying suction or rinsing, with the maximum temperature settings (100% power, xenon, Neuro-0) (Figure 4A), a slight temperature drop (~1–2°C) occurred after suction was initiated when the light remained on, while continuous suction (Figure 4B) demonstrated a more robust cooling effect than discontinuous suction (Figure 4C). The 10 mL saline rinsing at ambient temperature caused a precipitous temperature drop within 10 s, resulting in a temperature close to the baseline, and it took at least 2.5 min for the temperature to rise to about 37°C (Figure 4D).

## Discussion

In the EES, two-handed surgery might be useful depending on the surgery. Although the one-handed technique could be feasible in middle ear ossicular chain reconstruction, the two-handed technique might result in better exposure and control of the bleeding while resection of a glomus tympanicum or bleeding lesions. The surgeons also have to make a balance among exposure, workspace, and surgery safety in EES, though smaller diameter (e.g., 3 mm) endoscopes lead to less heat, larger diameter (e.g., 4 mm) endoscopes might lead to better exposure. A good vision of anatomical structures is mandatory for otological surgery. Therefore, the choice of endoscope diameter is guided by the external auditory canal size allowing one to work with one tool in one-handed EES or two tools with a two-handed EES. Then, a compromise of 3.3 mm endoscope was selected, and its 25 cm in length (neuro-endoscope) happens to meet the design and need of RobOtol®, as reported recently (9). It is reasonable to presume that with the progress in camera and image processing technology, the endoscope in the future will be even thinner with a preserved excellent image quality.

In the present study, we focused on the thermal safety of neuro-endoscopes for robot-assisted EES, to address concerns about heat issues among doctors who have not yet used the RobOtol® or will use the RobOtol® to assist in complex surgery. In EES, Bottrill et al. (22) first reported a temperature rise in the lateral semicircular canal with oto-endoscope applying. They recorded a maximum temperature of 55°C 2 mm in front of the tip of the endoscope, which could result in burns and charring. MacKeith et al. (14) reported that the tip temperature rose to 67.4°C, indicating the importance of avoiding the tip directly touching tissue. Previous similar reports did not investigate the effects of a range of power settings, and temperature measurements were usually limited to the endoscope tip (3, 17–22). Routine oto-endoscopes were introduced in first part of this study to provide a comprehensive comparison.

As expected, neuro-endoscopes, which have a larger diameter, result in more heat and light being applied to the illuminated area than oto-endoscopes with a similar light source and power settings. These findings strongly suggest that attention should be paid to the power settings and heat diffusion of neuro-endoscopes used with robotic assistance. Although the full range of output settings are rarely applied in the clinic, even at < 50% power setting, which might be applied in many centers (12, 17, 19), there would be thermal damage to the inner ear. Under 50% power setting, Das et al. reported (24) that merely converting the type of light source from LED to xenon will cause a higher temperature rise and significantly deteriorate the higher-frequency hearing. Therefore, based on our results, it can be reasonably inferred that an endoscope with a larger diameter transmits more heat, which will inevitably increase the severity of damage to delicate structures such as the inner ear. We also further explored the safe working distance. The suggested safe working distance for these neuro-endoscopes was 1 cm in front of the tip. Only for Neuro-30, the distance could be 0.5 cm in front of the tip when using LED power. Future *in vivo* studies or clinical experience with robotic system might be helpful and valuable to confirm this distance. Through the application of navigation and robot system software optimization, safe distances that are difficult to maintain by manual operation can be easily achieved with robot assistance. Unexpectedly, a rapidly increasing output was observed with increasing power using a xenon light with 375 h usage times between 45 and 50% power, and a less sharply increasing output was observed when using a xenon light with 128 h usage times. This phenomenon could be attributed to the way that light intensity is adjusted using the shading plate. The LED source showed a more stable output with power and may be a better choice than a xenon source in clinical practice (12, 24).

For the same endoscope, the profiles of light intensity and endoscope tip temperature were similar over the entire light source power settings, and light intensity was correlated with endoscope tip peak temperature. This correlation makes it possible to predict the maximum endoscope tip temperature and evaluate the functional status of the light sources over the entire power range in a short time by measuring the light intensity, which could be used as a routine self-check process for robot-assisted EES. In addition, the power setting of a given temperature could be estimated, while future research should be performed to investigate which maximal temperature at the tip is safe, for EES. Furthermore, the correlation could be used to establish a temperature estimation model to predict the stabilized temperatures of other combinations of endoscopes, light sources, and fiber optic cables under certain conditions.

White balance adjustment is generally applied before surgical manipulation. This will decrease the brightness on the screen but not in the surgical cavity. As each surgeon has his own preference for power setting in EES, the surgeon or operation room nurse should reduce the power setting when using larger and longer endoscopes, not just when adjusting white balance.

Suction and rinsing, which are regularly used in clinical practice, could give varying degrees of cooling effects. Rinsing was found to be much more effective. In cadaveric human temporal bone (36°C chamber, xenon and LED, 100 or 50% power), Kozin observed the maximum temperature at the round window membrane and rose by 8–10°C relative to the chamber temperature, and the cooling effect while applying a #20 French suction resulting in an 11°C temperature drop. However, #20 French suction (nearly ~6.7 mm in diameter) was not applicable to routine EES, which led to the choice of a #3 French suction (1 mm in diameter) in the present study. Accordingly, cooling measures such as continuous suction or intermittent rinsing (10 mL ambient temperature saline every 2.5 min or so) should be integrated into the robotic system applied in clinical practice to ensure safety during EES.

The main weakness of our study was *in vitro*. Thermal features are different *in vivo* (22). The lower temperature gradient *in vivo* may reduce the cooling rate because, *in vivo*, the surrounding temperature would be 37°C, or perhaps slightly lower, due to anesthesia and operating room temperature, but obviously not as low as 25°C as in this study. An active circulation system would dissipate some of the heat. In addition, the higher humidity and reflective properties of the tissue would mitigate temperature fluctuations.

Another disadvantage that should be noted is that the two-handed EES is not always feasible in a limited external auditory canal whose smallest maximum diameter range from 6.5 to 15.0 mm and the smallest minimum diameter range from 3.4 to 6.4 mm (25). In our clinical experience, a pure transcanal approach with two tools and a robot-held endoscope is difficult in external auditory canals narrower than 6 mm. This is because the endoscope is 3.3 mm wide and we use common otological tools. There may be no additional benefit from insisting on using the RobOtol® technique throughout the procedure under current technology. The steps that are easier with two tools are the tympano-meatal flap dissection (blood suction and flap pulling and tension), the tympanic membrane dissection from the malleus handle, the partial or total prosthesis placement, and the graft (cartilage or fascia placement). All other steps can be done with one hand for sure. We recommend starting using the robot-held endoscope with a large auditory canal during the learning curve. With a smaller endoscope or dedicated tool (in diameter and bayonet shape), we may be able to work in smaller canals but these tools are ready yet.

Yet despite these limitations, we believe our findings of light intensity and temperature changes with power, time, and cooling measures may be useful in clinical practice. Furthermore, the results for thermal spread from the tip of the endoscope and thermal distribution in human temporal bones may help in make EES practice as safe as possible. We might not need to use more power to obtain high-quality images as images taken during surgery by routine oto-endoscope at lower light intensities have no loss of quality (26), and future studies should investigate the light intensity applied during surgery and verify the functional consequences of the corresponding temperatures in an animal model.

The clinical application of robotics is a general trend, and related technologies will develop rapidly in the foreseeable future. Similarly, endoscopic imaging technology will continue to improve. The advancement of endoscopy technology may be able to fundamentally solve the problem of excessive temperature elevation caused by endoscope application by reducing the heat generation of the light source with the next-generation cold light source and improving imaging quality at low light intensity. Meanwhile, the advancement of micro-sensing technology could make the robot's perception more sensitive, and realize the real-time monitoring of the ambient temperature under the endoscope application, which provides further safety guarantee for the robot-assisted EES. Further, inspired by the single-port transoral robotic surgical system (27), subsequent development of robotic EES could further miniaturize relative devices to make the utmost of the middle ear space. Then, in single-port trancanal robotic EES, almost all the middle ear cavity is visible, and multiple-manipulator-operation could be achieved throughout the surgery. Before that, endoscope holder/robotic arm design should still take into account our findings of light intensity and temperature variations with light source power setting, and the temporal and spatial distribution of temperatures.

## Conclusions

Under the same conditions of the light source and power setting, neuro-endoscopes produce more heat than oto-endoscopes. LED light sources are associated with less significant temperature rises and have a more stable output with increasing power than xenon light sources. The light intensity at the endoscope tip could predict local temperature for a given endoscope and light source. Suction results in a slight temperature drop, while local rinsing is more effective in cooling. Applying submaximal light intensity, a LED source and intermittent rinsing should be considered for safer robot-assisted EES using a neuro-endoscope which allows a two-handed surgical procedure.

## Data Availability Statement

The raw data supporting the conclusions of this article will be made available by the authors, without undue reservation.

## Author's Note

This study was independent of the clinical trial of RobOtol®. Partially results of preliminary study were previously presented as a poster presentation at the 17th National Scientific Congress of Chinese Society of Otolaryngology-Head and Neck Surgery on November 27–29, 2020, online.

## Author Contributions

JS, ZW, OS, HJ, and HW: conceptualization. JP and HT: methodology. JP, HT, OS, and HJ: formal analysis. HW and OS: resources. JP: writing/original draft preparation. JS, ZW, OS, and HJ: writing/review and editing. ZW and HJ: funding acquisition. All authors contributed to the article and approved the submitted version.

## Conflict of Interest

The authors declare that the research was conducted in the absence of any commercial or financial relationships that could be construed as a potential conflict of interest.
